# Aptamers as the Agent in Decontamination Assays (Apta-Decontamination Assays): From the Environment to the Potential Application* In Vivo*

**DOI:** 10.1155/2017/3712070

**Published:** 2017-10-26

**Authors:** Mawethu Pascoe Bilibana, Marimuthu Citartan, Tzi Shien Yeoh, Timofey S. Rozhdestvensky, Thean-Hock Tang

**Affiliations:** ^1^Advanced Medical and Dental Institute, Universiti Sains Malaysia, 13200 Kepala Batas, Penang, Malaysia; ^2^Medical Faculty (TRAM), University of Muenster, Von-Esmarch-Str. 56, 48149 Münster, Germany

## Abstract

The binding specificity and affinity of aptamers have long been harnessed as the key elements in the development of aptamer-based assays, particularly aptasensing application. One promising avenue that is currently explored based on the specificity and affinity of aptamers is the application of aptamers in the decontamination assays. Aptamers have been successfully harnessed as the decontamination agents to remove contaminants from the environment and to decontaminate infectious elements. The reversible denaturation property inherent in aptamers enables the repeated usage of aptamers, which can immensely save the cost of decontamination. Analogous to the point-of-care diagnostics, there is no doubt that aptamers can also be deployed in the point-of-care aptamer-based decontamination assay, whereby decontamination can be performed anywhere and anytime for instantaneous decision-making. It is also prophesied that aptamers can also serve more than as a decontaminant, probably as a tool to capture and kill hazardous elements, particularly pathogenic agents.

## 1. Introduction

Environmental contamination has been a major concern worldwide for the past two centuries [1]. Major environmental contaminants are organic compounds such as small organic and inorganic pollutants, pharmaceuticals, personal care products, toxins of microbial origin, and pathogens [2–5]. These environmental contaminants have severe impact on quality of food, air, and water, in turn exerting mild to severe deadly effects on human and animals. A range of decontamination methods have been devised to remove environmental contaminants in contaminated soils, leachate, wastewater, and infected tissues [6–8]. General strategies used for removing contaminants from contaminated environment include chemical precipitation, ion exchange, reverse osmosis, and solvent extraction [9]. A novel strategy that is worth venturing into is to use aptamers to remove contaminants in contaminated environments [10, 11].

Generated by Systematic Evolution of Ligands by Exponential Enrichment (SELEX), aptamers are short synthetic oligonucleotides, either DNA or RNA, that specifically bind target molecule(s) with high specificity due to their specific and complex three-dimensional shape comprised stems, loops, bulges, hairpins, pseudoknots, triplexes, and quadraplexes [12–14]. Aptamers are able to bind a wide variety of targets from divalent metal ions, small organic molecules, proteins, and cells [15, 16]. Binding specificity and affinity of aptamers are harnessed in various applications, majorly in biosensing applications [10, 11, 17–26]. Apart from biosensing application, aptamers have also been the subject in the aptamer-based capture assay. In the capture assay applications, aptamers are applied for the purification of their corresponding targets for a wide variety of purposes [11, 27, 28]. The present review seeks to present an overview of the more narrowed context of the aptamer-based capture assay, which is on the usage of the aptamer in the decontaminating process or simply abbreviated as apta-decontamination assay. Several aspects of the apta-decontamination assay will be deliberated, which encompasses the functionalization of the aptamer to the usage of the aptamer in decontaminating environment, food sample, and in* in vivo* application. Reusability of the aptamer and the development of point-of-care apta-decontamination assays are explored before envisaging the potential future trend of the apta-decontamination assay.

## 2. Potentiation of the Apta-Decontamination Assays Is Fueled by the Availability of a Large Number of Small Molecule Aptamers

Most of the contaminants present in the environment are small molecules [29]. Despite the technical challenges in isolating aptamers against small molecules such as the target immobilization and the determination of binding affinities, a significant number of small molecule aptamers were isolated. The availability of a myriad of small molecule aptamers suggests that these aptamers can be used as the corresponding decontaminating agents [28]. One important property of the aptamer that substantiates its role as the decontaminating agent is the exceptional discerning ability of the aptamers even against small molecules. RNA aptamer against theophylline was able to discern against the closely similar molecule, caffeine, that differs from the former by just a methyl group [30]. The aptamer binds caffeine with a binding affinity weaker by 10,000 times compared to that of the theophylline. RNA aptamers were also able to distinguish between various L and D amino acids [31, 32].

## 3. Functionalization of Aptamers for Biodecontamination

**Figure 1:**
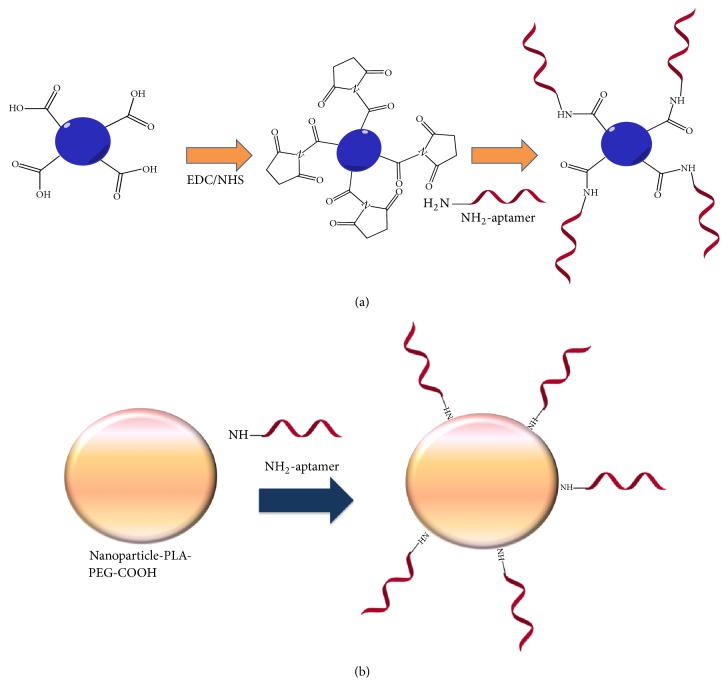
(a) Schematic representation of the conjugation of the amine-terminated aptamer on the surface of the carboxylated-functionalized sepharose 4FF beads via NHS/EDC activation [35]. (b) Conjugation of the amine-functionalized aptamer on the surface of nanoparticles that contain a stable poly (lactic acid)-polyethylene glycol (PLA-PEG) block copolymer with a terminal carboxylic acid functional group (PLA-PEG-COOH) [37].

Akin to the capture assay application as reviewed by Citartan et al. [27], the very first step for the application of the aptamers for the biodecontamination is the immobilization of the aptamers on the surface of the platforms. To enable this immobilization, aptamers are first functionalized with specific functional groups (e.g., thiol, amine, and biotin) [22, 33]. Interaction of these functional groups with the functional groups on the surface of the platforms immobilizes the aptamer. Typical functionalization strategy is by appending amine groups at the aptamer termini (5′ or 3′-end), which can form peptide bond with the carboxyl groups on the surface of the platform. The carboxylated surface is first subjected to N-hydroxysuccinimide (NHS)/1-Ethyl-3-[3-dimethylaminopropyl] carbodiimide hydrochloride (EDC) solution activation to form active NHS ester that can react with the primary amines. Peptide bond-mediated conjugation of the aptamer on the platform surface was adopted by Dong Huy et al. [34], in which the anti-17*β*-estradiol (E2) aptamers used were appended with primary amines. The glass microbeads were first amino-silane modified and then treated with phenylene diisothiocyanate (PDITC) to generate the isothiocyanate-modified bead, in which the active isothiocyanate group can react with the amine-functionalized anti E2 aptamer. Chen et al. [35] have fabricated a hepatitis C virus- (HCV-) specific aptamer conjugated with amine to react with the sepharose 4 Fast Flow (4FF) beads to immobilize the aptamer. The carboxylated-derivative sepharose 4FF beads, which is 4% highly cross linked spherical sepharose is activated via NHS/EDC prior to peptide bond formation with the amine-terminated aptamers (Figure 1(a)).

For immobilizing aptamers, nanoparticles are also employed as the platform, which have more surface area to volume ratio for the immobilization of aptamers [36]. Hu et al. [37] have conjugated amine-functionalized aptamer on the surface of nanoparticles that contain a stable poly (lactic acid)-polyethylene glycol (PLA-PEG) block copolymer with a terminal carboxylic acid functional group (PLA-PEG-COOH) (Figure 1(b)). The carboxylic acid functional groups form peptide bond with the primary amines. This immobilizes the aptamers, which were used for the decontamination of mercury (Hg^2+^)* in vivo*. It was also found out that Metal-Organic Frameworks (MOFs) as the platform also has high surface area that can accommodate more aptamers [38]. In one study, MOFs were generated which comprise Fe_3_O_4_ nanoparticles encapsulated with a polydopamine layer which was then functionalized on the surface with UiO-66-amine [39]. However, to form a covalent bond between the amine-conjugated aptamer and the UiO-66-amine, glutaraldehyde linker was used. The aptamer was applied for the decontamination of polychlorinated biphenyls in contaminated soil samples.

Another alternative to conjugate directly to the amine-conjugated aptamers is to use Cyanogen-bromide (CNBr) activated crosslinker. CNBr reacts with the hydroxyl group to generate cyanate esters or imidocarbonates, which can readily react with primary amines-containing molecules. Madru et al. [40] covalently immobilized anticocaine aptamer conjugated amine on CNBr-activated sepharose. However, there is growing concern associated with high toxicity of CNBr and its potentiality as an environmental pollutant [41]. In addition, surface immobilization of CNBr is time consuming, which may inactivate the biological activity of aptamer. Biotin-streptavidin interaction is also used for aptamer immobilization although the interaction is less strong than the peptide bond. However, the biotin-streptavidin interaction is rapid and stable over a wide range of pH values and temperatures [42]. The isoelectric point of streptavidin is 6.8 to 7.5, which minimizes nonspecific adsorption within this pH range. Biotinylated arsenate (As(V/III)) aptamers functionalized on the surface of streptavidin agarose resin were used to completely remove As(V/III) in the aqueous solution [43]. After the completion of the aptamer immobilization via all the strategies, the unconjugated aptamers should be removed from the immobilization surface. This is important as carry-over of these unreacted aptamers into the “target-capturing process” may result in nonconjugated aptamer-target formation. If this happens, the decontamination capacity of the immobilized aptamers is reduced.

## 4. Aptamers as the Pollutant Absorbent in the Environment

**Figure 2:**
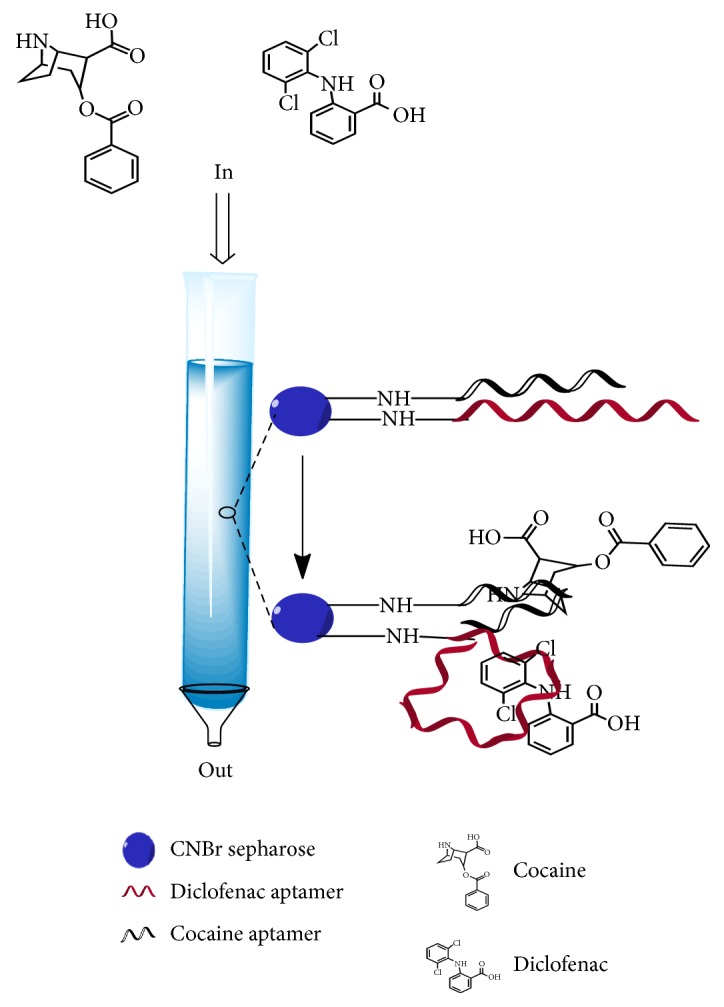
Illustration of apta-decontamination of cocaine and diclofenac. In this assay, aptamer against cocaine and diclofenac are immobilized on the resin within a column for the decontamination of cocaine and diclofenac [46].

Decontamination process is traditionally achieved by chemical precipitation, ion exchange chromatography, and other methods [9]. However, these are inexpensive and it is not feasible to reduce their concentrations to the levels as low as that required by the environmental legislation [44, 45]. A very wise alternative is to use aptamers as the pollutant absorbents, for binding and removing the environmental contaminants. Moreover, aptamer-based adsorption does not result in any secondary pollution as no harmful substances are produced during the process. One example of aptamer used in the apta-decontaminant assay is the DNA aptamer against arsenite [As(III)] and arsenate [As(V)]. Kim et al. [43] developed a novel apta-decontamination method for the complete removal of 28.1 to 739.2 *μ*g L^−1^ As(III) and As(V) from the groundwater after just 5 min of incubation with its corresponding aptamer. Complete removal of the target can be achieved in a very short time due to the fast on and off binding kinetics of the aptamer with the target. In a similar manner, Hu et al. [46] have developed an apta-decontamination assay that is able to decontaminate organic contaminants such as cocaine and diclofenac at ng L^−1^ levels in drinking water (Figure 2). In fact, As(III) and As(V) aptamer column have a better decontamination efficiency compared with the other filtration technology as demonstrated by Hsieh et al. [47] on their electro-ultrafiltration system. The electro-ultrafiltration system has a cut-off value of 100 kDa and requires the use of electrical voltage of 25 V to achieve As(III) and As(V) decontamination efficiency of 79% in groundwater samples [43]. It is manifested that the apta-decontamination assay requires no electricity and is more specific than the nonspecific nature of the ultrafiltration system that relies on molecular-weight cut-off value to decontaminate target.

Ion exchange system is based on the reversible chemical reactions for removing dissolved ions from solution and replacing them with other similarly charged ions [48, 49]. It is timely to mention that, due to higher selectivity and affinity of the aptamer against the target ion, apta-decontamination assay could potentially replace or complement ion exchange system in decontamination. As a proof-of-concept, aptamer against uranyl ion was used for the decontamination of uranium. Uranium contaminated drinking water is a common problem resulted from radioactive disposal waste [50]. Kim et al. [51] have immobilized thiolated aptamer against uranyl ion on the surface of the sulfo-SMCC (sulfosuccinimidyl 4-(N-maleimidomethyl)cyclohexane-1-carboxylate)-activated aminopolystyrene resin as the solid support. The decontamination assay is able to decontaminate 0.63 mg of uranium amidst the presence of the other multiple ions in the sample [51]. Aptamer as the decontaminating agent is also able to discriminate between closely similar molecules and is very selective against the target. Recent study has shown the RNA aptamer that selectively adsorbs traces (ng L^−1^) of microcystin-LR (MC-LR) in drinking water [52], discriminating against microcystin-RR, microcystin-LW, and nodularin which are cyclic pentapeptides that have a similar structure with MC-LR. This aptamer could be used as the decontaminating agent to remove MC-LR. MC-LR is a potential carcinogen for animals and humans that is present in aquatic environment [53, 54].

**Figure 3:**
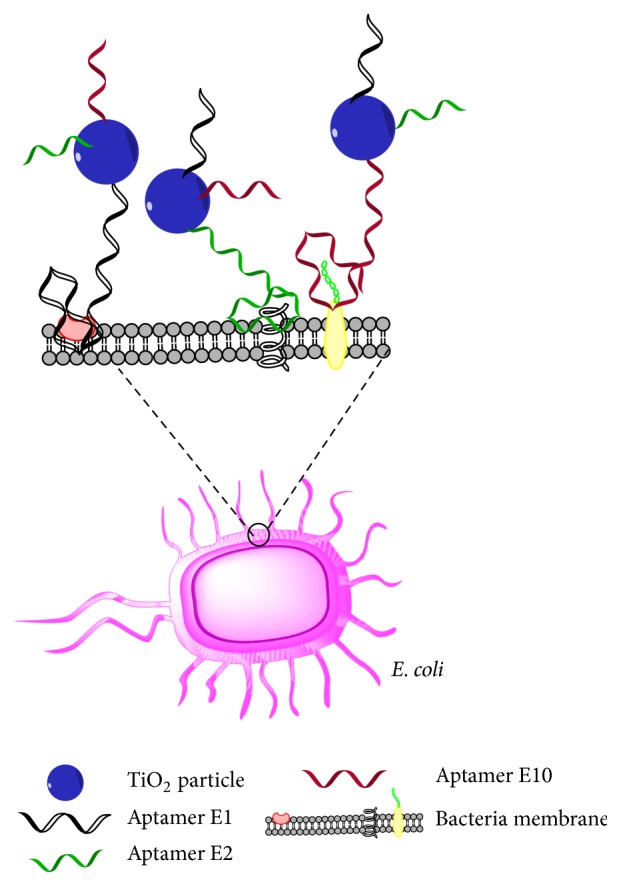
Schematic diagram of apta-decontamination of bacteria. Three aptamers specific against three different bacterial surface proteins were immobilized on surface of TiO_2_ to specifically capture *E. coli*, which is then deactivated under UV irradiation [56].

Isolation of aptamers against bacterial surface proteins or against the whole-cell [55] permits their application in removing bacteria from the environment. Song et al. [56] used three different aptamers selected using a bacterial cell–SELEX that have different affinities and target different proteins on surface of* Escherichia coli (E. coli)*. The target proteins may be components such as Lipopolysaccharide (LPS), outer membrane proteins, and flagella, which must be confirmed by aptamer-facilitated biomarker discovery (AptaBiD) [57]. These amino-modified aptamers were immobilized on the surface of TiO_2_ for targeted and enhanced disinfection of* E. coli* (Figure 3). The target* E. coli* was approximately 99% inactivated under UV irradiation in 30 min. The assay was also specific and selective, as it inactivates only* E. coli* in the presence of a mixed culture* (E. coli and Staphylococcus epidermidis)*. Besides bacteria, aptamers were also used to remove contaminating bacteria toxins from soil, food, and water environment [9, 58].

**Figure 4:**
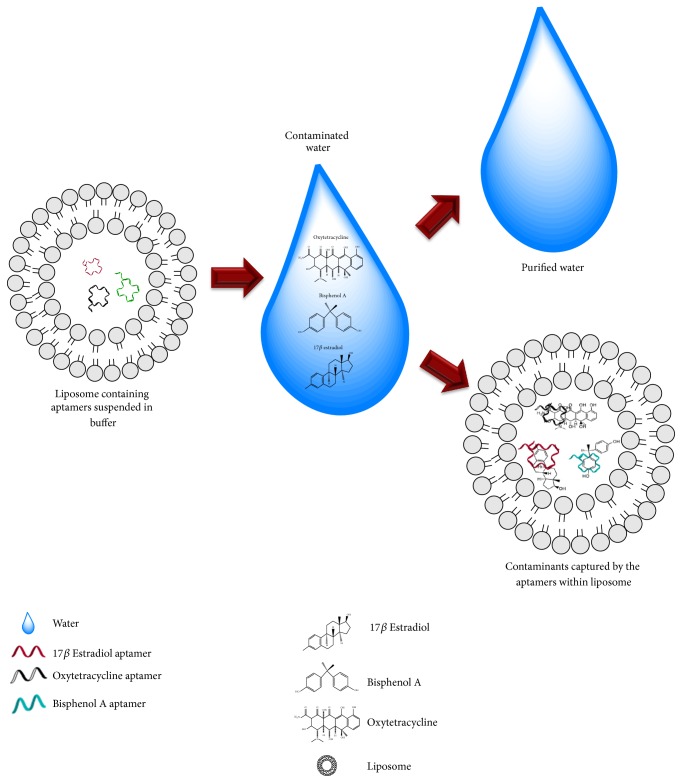
Illustration of apta-decontamination using liposome containing aptamer. Liposomes that contain the aptamer binding buffer also comprise aptamers that are specific against 17*β*-estradiol (E2), bisphenol A, and oxytetracycline. These aptamers bind the contaminants (17*β*-estradiol (E2), bisphenol A, and oxytetracycline) and remove them from the contaminated water source [59].

In general, a cause for concern is the difference in the ionic strength/pH of the aqueous environmental target from the optimal buffer used for the aptamer interaction that could debilitate its binding affinity. To address this problem, aptamers should be placed in a distinct compartment to thwart them from being affected by surroundings that will undermine their binding capacity. As such, liposomes that contain the aptamer binding buffer could be possibly used as the holding moiety for aptamers. Kim et al. [59] have constructed biocomposite material that comprise liposome that contains aptamers dissolved in the binding buffer for the selective and simultaneous decontamination of E2, bisphenol A (BPA), and oxytetracycline from the contaminated water within 30 minutes (Figure 4). The capturing efficiency is higher (more than 80%) when liposomes are used as the holding moiety as compared to the DNA aptamers in water. The cases enumerated are sufficient to imply that the apta-decontamination assay could potentially replace or complement the current existing strategies in decontaminating small molecules in the environment.

## 5. Apta-Decontamination Assay on Food Sample

Besides, majorly in the environment, the capturing capacities of the aptamers are also exploited to remove contaminants from food sample. Most of the apta-decontamination assays on food sample reported so far revolve only around ochratoxin A (OTA) aptamer, isolated by Cruz-Aguado and Penner [60], with the estimated dissociation constant (*K*_*d*_) of 360 nM. They have developed the first aptamer-based affinity column for the concentration and decontamination of OTA. The OTA aptamers were conjugated to agarose resin and were packed into a pipette tip, which was used as the column. The performance of the affinity column-functionalized aptamer was first tested with a buffer spiked with OTA, whereby more than 97% of OTA was removed from 1 mL of a 100 nM OTA solution. Subsequently, the apta-decontamination assay of OTA was further coupled with fluorescence detection for the better monitoring of the OTA removal. In this study, Yang et al. [61] developed an aptamer affinity column with ultrahigh performance liquid chromatography coupled with fluorescence detection to remove OTA from ginger powder. The amount of OTA removed from the ginger powder ranged from 1.51 to 4.31 *μ*g kg^−1^, which was lower than the European Union regulatory limits. Average recovery of 85.36–96.83% was acquired for blank samples spiked with OTA at 5, 15, and 45 *μ*g kg^−1^.

The OTA removal was also facilitated with the use of magnetic field mediated by magnetic nanoparticles, which can offer higher capturing efficiency with shorter diffusion time. Wu et al. [62] have immobilized OTA aptamers on the surface of the magnetic nanospheres and have developed a magnetic solid phase extraction procedure to remove OTA from the food samples. The assay developed consists of high performance liquid chromatography separation and a fluorescence detection (HPLC-FD) system [62]. The clean-up of the OTA was facilitated by magnetic field and the removal of OTA was monitored by HPLC-FD. With 2.5–50 *µ*g kg^−1^ of OTA spiked in different samples of cereals products and wheat flour spiked, the recoveries varied from 67 to 90%. The apta-decontamination assay showed a high selectivity in comparison to a conventional hydrophobic sorbent, with a good extraction recovery. However, as observed in the case study by Schax et al. [10], using OTA, the binding capacity of the aptamer against the corresponding targets is diminished in complex sample. Thus this strongly suggests that during* in vitro* selection, complex media could be used as the SELEX binding buffer. The complex media, which are optimal for the aptamer performance, will ensure that the aptamer binding is retained during the decontamination process. Moreover, Sefah et al. [63] have shown that the* in vitro* selection carried out in culture media Dulbecco's phosphate-buffered saline as the SELEX binding buffer is still be able to produce aptamers that bind target in the similar buffer.

Apta-decontamination system enables specific extraction of toxins or natural compounds from turbid matrices in a one-step procedure. Rouah-Martin et al. [64] have developed apta-decontamination of ergot alkaloids (causing severe poisoning known as ergotism), using DNA aptamer-based ergot alkaloids immobilized on silica for specific solid phase extraction system. The assay enables specific extraction of ergot alkaloids from a contaminated rye feed sample prior to the analysis of the extract using liquid chromatography quadrupole-time-of-flight mass spectrometry. The success manifested by the OTA and ergot alkaloids aptamers may further fuel the isolation and subsequent application of the aptamers against contaminants in the food product.

## 6. Aptamers as the Potential* In Vivo* Decontamination Agent

High specificity conferred by aptamers with minimal off-target binding accounts for its wide application in the fields of drug delivery, therapeutic agent, molecular biosensing, and biomimetic engineering [17, 65–70]. This also strongly insinuates that aptamers can also be potentially used to decontaminate contaminants* in vivo*  [27, 35].

Aptamer-based decontamination assay is used to selectively capture and detoxify infectious agents. In one study, Chen et al.[35] used HCV-specific single-stranded DNA aptamers to decontaminate approximately 80% of the HCV from human plasma samples. This novel apta-decontamination procedure could effectively remove HCV particles and likely to serve as a novel therapy option for HCV. Proske et al. [71] have used nuclease-resistant 2′-amino-2′-deoxypyrimidine-modified RNA aptamers (DP7) that recognize a peptide comprising amino acid residues (90–129) human prion protein with high specificity. This particular region is important for the conversion of PrP(C) into its pathogenic isoform PrP(Sc). This aptamer results in the reduction of PrP(Sc) accumulation in prion-infected cells. The DP7 aptamer may open the door towards using aptamers as a rational therapeutic of transmissible spongiform encephalopathies.

**Figure 5:**
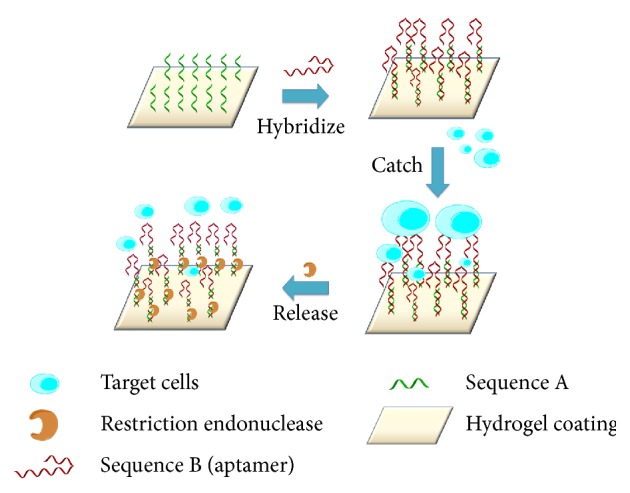
Schematic diagram of an application of aptamer in capturing and releasing tumor cells. Sequence immobilized on the surface of the hydrogel forms a duplex with an aptamer sequence. Cleavage of the restriction sites on the aptamer (sequence B) and the immobilized sequence (sequence A) releases the target cells. Endonuclease cleaves the phosphodiester bond within the aptamer, resulting in the loss of its anchoring to the target cells. Subsequently, the target cells are dissociated from the aptamer and released [72].

The high stability and less immunogenicity associated with aptamers potentiate aptamers for the selective capturing and eliminating a circulating tumor cells. Li et al. [67] presented a study that makes use of hydrogel functionalized with two layers with the top one containing DNA aptamer specific against human precursor T-cell acute lymphoblastic leukemia cells (CCRF-CEM), while the bottom one contains double-stranded DNA as the “affinity tag” for the intercalation of doxorubicin to eradicate target circulating tumor cells. The aptamer on the top hydrogel layer was able to capture CCRF-CEM cells with high efficiency and specificity, while the doxorubicin sequestered at the bottom is released to kill tumor cells. Moreover, Li et al. [72] developed an endonuclease-responsive circulating tumor cells aptamer that was functionalized on hydrogels for the specific catch and nondestructive release of rare circulating tumor cells. The aptamer was hybridized to another sequence that is immobilized on the surface of the hydrogel. Both the sequence immobilized on the surface of the hydrogel and the aptamer sequence harbour restriction endonuclease cleavage site, targeted by* BamHI*. The cells captured by the aptamers will be released after the cleavage of the restriction sites (Figure 5). Specificity of the assay is also substantiated as the assay developed showed binding of CCRF-CEM cells rather than Ramos cells on the hydrogel surface. The capture and release of circulating tumor cells can be used for decontamination and for the detailed analysis of circulating tumor cell properties [72]. Reports found out that chemotherapeutic agents such as cisplatin or 5FU increase the amount of heat shock protein 70 (HSP70) exosomes, which then activates myeloid-derived suppressive cells (MDSCs) obstructing the development of an antitumor immune response [73]. Exosomes through HSP70 expressed in their membrane then interact with the toll-like receptor 2 (TLR2) to activate MDSCs. Gobbo et al. [74] reported the capturing application of the peptide aptamer that binds to the extracellular domain of membrane HSP70 and to capture HSP70 exosomes from cancer patient samples. The aptamer is able to block the interaction between HSP70 and TLR2, suppressing the activation of the MDSCs. In fact, aptamers can be a promising class of molecules for decontaminating contaminants* in vivo* owing to high selectivity and their superior characteristics of high cell penetration capacity.

## 7. Reusability of Aptamers as the Decontaminating Agents

**Figure 6:**
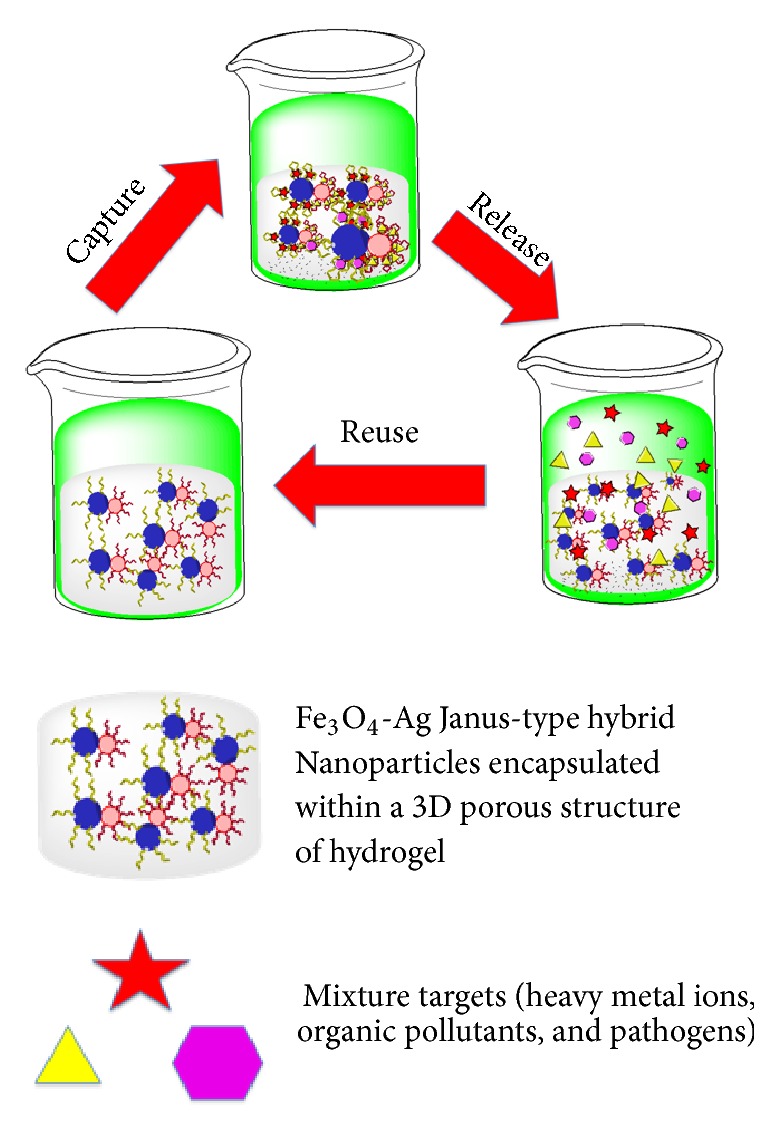
Schematic representation of the reusability of aptamer-based hydrogel scavenger in apta-decontamination of three pollutants. Aptamers are immobilized on the surface of the Fe_3_O_4_-Ag Janus-type hybrid nanoparticles encapsulated within a 3D porous structure of hydrogel. The targets (heavy metal ions, organic pollutants, and pathogens in drinking water) are captured by the aptamers. Aptamers are reused/regenerated by changes in the pH and the aptamers can be reused for the subsequent capture assay [77].

An ideal criterion of a decontamination assay is reusability, which can be conveyed by aptamers that have reversible denaturation property [75, 76]. Reusability of the aptamer refers to the repetitive usage of aptamers in binding and eluting their corresponding targets with minimal loss of capturing efficiency. Aptamers can be denatured and renatured to their original conformations by changing the temperature and pH. Recently, Hu et al. [46] appraised the reusability of an aptamer-based column in removing cocaine and diclofenac from the drinking water. Decontamination capacities of the aptamers were evaluated before and after 30 days at 4°C and were found to decrease by only 1.2–4.7%. Moreover, the regeneration of the aptamer-based column was achieved by simple washing with pure water at a rate of 10 mL min^−1^ at 50°C for 5 min. The decontamination capacity of the aptamer column decreased approximately by 10% after 10 regeneration cycles, corroborating that covalent binding between aptamers and CNBr-sepharose is stable despite using hot water. Using a similar approach of regeneration, Hu et al. [52] have “regenerated” MC-LR aptamer functionalized on surface of RNA–graphene oxide with hot water and their apta-decontamination capacity reduced by about less than 10% after 5 cycles. Schax and coworker [10] tested the reusability of OTA aptamer-based column for 4 additional cycles of decontamination within 20 days. In between the decontamination cycles, the aptamer-based column was washed with binding buffer to ensure refolding of the aptamer followed by storage at 4°C in binding buffer. The aptamer remained stable for a period of 20 days, and the amount of OTA obtained did not change profoundly, between 84 and 94% for all the cycles, with elution efficiency between 71 and 84%. Wang et al. [77] reported *a* hydrogel scavenger containing aptamers immobilized on Fe_3_O_4_-Silver (Ag) Janus-type hybrid nanoparticles encapsulated within a 3D porous structure of hydrogel for high efficiency of simultaneous decontamination of Hg^2+^ (99.96%), BPA (98.9%), and* E. coli* (100%) in drinking water as showed in Figure 6. Regeneration is achieved based on the changes in the pH, whereby the hydrogel scavenger was washed with HCl (pH 1) and water three times. In almost all the cases mentioned, the efficiency of the aptamers remains the same after several cycles of decontamination, which demonstrates the excellent reusability property of the aptamer. Another elution strategy is based on the usage of the chelating agent, which chelates the divalent ions that are important for the binding affinity of the aptamer against the target. In the presence of the chelating agent that chelates the divalent ion, the target is released from the aptamer. This is because the aptamer is unable to retain the structural conformation without the presence of the divalent ion. In one surface plasmon resonance analysis that aims to look at the significance of the calcium (Ca^2+^) ion for the interaction between the RNA aptamer and its corresponding target human immunoglobulin (IgG1), addition of ethylenediamine tetra-acetic acid (EDTA), which chelates the Ca^2+^, results in the loss of the binding affinity between these two molecules. However, the affinity is regained upon the addition of the ion. It can be surmised that the chelating agents can be used for the elution of the target [78]. Integration of the aptamer into the decontamination assay can immensely save cost. The overall cost of manufacturing aptamer is plummeting, which is also influenced largely by the increasing demand for oligonucleotides that accounts for the reduction in the cost of nucleotide phosphoramidites. For one gram of DNA phosphoramidites, the estimated cost is less than US$3, while that for per gram of 2′-fluoro, 2′-ribo, and 2′-O-methyl RNA phosphoramidites costs less than US$20 [79].

## 8. Chemical Modifications for the Enhanced Stability of the Aptamer

During the apta-decontamination process, the most prominent challenge is to stabilize or protect the aptamers (especially RNA aptamers) from the degradation action of the nucleases that are present in the environmental soil and water. Stability can be achieved via modification of the aptamer following isolation by SELEX. However, due diligence should be practiced as POST-SELEX modification may impair the binding affinity of the aptamer against the target [80]. Hence, it is desirable to incorporate the chemical modification into the library used for the* in vitro* isolation of the aptamers. Modification includes the addition of cap at the 3′- and/or 5′- ends to occlude the exonuclease degradation action [81]. Stability of the aptamers can also be enhanced by using phosphorothioate nucleotides [82]. Another modification is Locked nucleic acids (LNAs), whereby the 2′-O is linked to the 4′-C of the ribose via a methylene bridge, giving rise to 3′-endo conformation that is stable against the action of nucleases [83, 84]. A highly nuclease-resistant aptamer, Spiegelmer, a mirror-image aptamer built from nucleotides of the nonnatural L-chirality, can be a perfect candidate for decontamination. As these L-aptamers are mirror images of the natural D-aptamer counterpart of the same sequence, the former is unrecognized by the stereoselective nucleases. Consequently, spiegelmers are highly resistant to the action of nucleases [85]. Modification is also achievable by substituting hydroxyl group at the 2′-position of the ribose sugar with the amino (NH_2_), fluoro (F), alkyl, and thio groups [86].

## 9. Point-of-Care Aptamer-Based Decontamination Assay

The diagnostic technology has moved into another dimension, whereby the diagnostic can be performed anywhere with a fast turnaround time that enables quicker treatment, known as the point-of-care testing [87]. Analogous to a point-of-care diagnostic assay, an apta-decontamination assay should also be developed on a similar basis, so that the decontamination can be carried out anytime that could permit instant decision-making. Fueled by the need to avoid health hazards, ecosystem, food, and water contaminated by a variety of microbial pathogens, toxins, heavy metals, organic pollutants, and adulterants should be subjected to decontamination before reaching consumers [88]. The ideal point-of-care aptamer-based decontaminating device should be of low power, low cost, reusable, provide durability, and have a high apta-decontamination capacity without any secondary pollution in purifying environmental pollutants. A point-of-care assay developed by Wang et al. [77] exhibited the ideal application of a novel apta-decontamination, which consists of an empty filter unit of a commercially available water purifier. The water purifier is packed with hydrogel scavenger that accommodates multiple aptamers that can capture pollutants. The hydrogel scavenger also holds Fe_3_O_4_-Ag Janus nanoparticles functionalized with multiple aptamers to remove metal ions, organic pollutants, and pathogens simultaneously. This apta-decontaminant showed an outstanding performance in reducing Hg^2+^ concentration from 1 ppm to levels lower than 1.1 ppb and BPA from 60 ppm to level smaller than 0.8 ppm and can effectively destroy more than 99.998% of* E. coli *simultaneously. This aptamer-based decontamination device can remove three different elements simultaneously, suggesting that multiple aptamers can be used in future for the removal of a myriad of major environmental contaminants. The study carried out could provide an impetus to expedite the development of point-of-care apta-decontaminating device that is mobile and with a platform that is immobilized with multiple aptamers that are specific against different targets.

## 10. Conclusion and Future Perspective

Despite great promise, there are major barriers that must be transcended to use aptamers as decontamination agents. Biostability of aptamers is one of the concerns that currently limits the application aptamers as decontamination agents in the real world [89]. Therefore, to generate aptamers with proved biostability, a direct evolution of a nuclease-resistant sequences from a library of different chemical substituents should be carried out [90, 91]. Another critical factor that must be heeded is the compatibility of the aptamer with the environmental condition which the aptamer is subjected to during decontamination process. Metal cation concentration, buffer conditions, and temperature are the main elements that influence the stability and affinity of the aptamers [92, 93]. The best approach is to mimic the actual environmental condition by which the aptamer is exposed to at the point of capturing process in the SELEX process itself. As such, aptamers that can optimally perform during the capture process can be successfully isolated [94].

Aptamers are quite flexible in the functionalization, thermodynamically stable, and able to bind target within a short period of time and are also stable despite repetitive usage. Endowed with these excellent properties, aptamers have “fared well” in decontaminating environment, food sample, and removing infectious agents, which is also supported by the availability of aptamers against a number of small molecular contaminants. Thus, it is felicitous to say that the apta-decontaminating assay should not merely be applicable for decontamination, but also be focused towards capturing and removing hazardous agents. One case study is exhibited by Ranjbar and Hafezi-Moghadam [95] who have designed a MPT64 antibody aptamer-based DNA nanorobot. The lock is constituted by the duplex formation between the aptamer sequence and the complementary strand. In the presence of the target, the aptamer strand binds to it, opening up the lock, releasing the drug molecules from the nanorobot that can interact with the bacterial surface protein. It is worth noting that DNA origami structure, which is a long single-stranded scaffold that could be moulded into any intended configurations using short ssDNA as the connectors [96, 97], could serve as a powerful smart material in the future that could assist the aptamers to capture and eradicate bacteria as well as virus-infecting eukaryotic cells. Inspiration can also be derived from Douglas et al. [98] who have fabricated DNA nanorobot by using DNA origami technology that is programmed to independently recognize target defected cells and deliver payloads. Aptamers specific against any targets can be infiltrated into the DNA origami and the whole ensemble can be adopted for targeted elimination of the bacteria. The application of DNA origami can be extended towards the simultaneous decontamination of a panel of contaminants in water via the usage of the corresponding specific aptamers. Multiple aptamers can be conjugated to the ssDNA constituents of the DNA origami and binding against their cognate targets may induce conformational change that can facilitate capturing and removal of the target.
